# Potato Non-Specific Lipid Transfer Protein StnsLTPI.33 Is Associated with the Production of Reactive Oxygen Species, Plant Growth, and Susceptibility to *Alternaria solani*

**DOI:** 10.3390/plants12173129

**Published:** 2023-08-31

**Authors:** Carol Bvindi, Kate Howe, You Wang, Robert T. Mullen, Conner J. Rogan, Jeffrey C. Anderson, Aymeric Goyer

**Affiliations:** 1 Department of Botany and Plant Pathology, Oregon State University, Corvallis, OR 97331, USA; carol85bvindi@gmail.com (C.B.); howekath@oregonstate.edu (K.H.); roganco@oregonstate.edu (C.J.R.); jeff.anderson@oregonstate.edu (J.C.A.); 2 Department of Molecular and Cellular Biology, University of Guelph, Guelph, ON N1G 2W1, Canada; wyou@uoguelph.ca (Y.W.); rtmullen@uoguelph.ca (R.T.M.)

**Keywords:** apoplast, biotic, lipid transfer protein, superoxide, *Alternaria*, potato

## Abstract

Plant non-specific lipid transfer proteins (nsLTPs) are small proteins capable of transferring phospholipids between membranes and binding non-specifically fatty acids in vitro. They constitute large gene families in plants, e.g., 83 in potato (*Solanum tuberosum*). Despite their recognition decades ago, very few have been functionally characterized. Here, we set out to better understand the function of one of the potato members, *StnsLTPI.33*. Using quantitative polymerase chain reaction, we show that *StnsLTPI.33* is expressed throughout the potato plant, but at relatively higher levels in roots and leaves compared to petals, anthers, and the ovary. We also show that ectopically-expressed StnsLTPI.33 fused to green fluorescent protein colocalized with an apoplastic marker in *Nicotiana benthamiana* leaves, indicating that StnsLTPI.33 is targeted to the apoplast. Constitutive overexpression of the *StnsLTPI.33* gene in potato led to increased levels of superoxide anions and reduced plant growth, particularly under salt stress conditions, and enhanced susceptibility to *Alternaria solani*. In addition, *StnsLTPI.33*-overexpressing plants had a depleted leaf pool of pipecolic acid, threonic acid, and glycine, while they accumulated putrescine. To our knowledge, this is the first report of an nsLTP that is associated with enhanced susceptibility to a pathogen in potato.

## 1. Introduction

Plant nsLTPs are small proteins (usually below 10 kDa) capable of transferring phospholipids between membranes and binding non-specifically fatty acids in vitro [1,2,3,4]. They have a conserved eight cysteine motif (8CM) with the general pattern C-Xn-C-Xn-CC-Xn-CXC-Xn-C-Xn-C [1,2,5]. They also possess four or five α-helices that are stabilized by four conserved disulphide bridges [3,5,6,7]. Genome analysis shows that they constitute large families of proteins in plants, e.g., 49 in Arabidopsis, 52 in rice, and 83 in potato, and are classified into different types according to the spacing pattern between cysteines of the 8CM, the amino acid sequence identity, the presence and position of introns, and glycosylphosphatidylinositol modification site [8,9,10,11]. Most plant nsLTPs have a predicted signal peptide for targeting to the extracellular space, and several studies have proven experimentally their extracellular, cell wall, or plasma membrane localization (for review, see [11]). They also can accumulate in high amounts in plant tissues; e.g., they constitute 4% of soluble proteins extracted from maize seedlings [12].

Although the function(s) of most plant nsLTPs remain unknown, in large part due to the absence of a phenotype in knock-out or knock-down mutants, several studies have shown their involvement in plant growth and development, as well as roles in plant resistance to abiotic and biotic stresses [11], whereby they are often referred to as pathogenesis-related (PR) proteins of the PR-14 family [13]. nsLTPs also have antimicrobial activities against bacteria and fungi [14,15,16] and play a role in long-distance systemic acquired resistance (SAR) signaling against bacterial infections [17,18]. Notably, the constitutive overexpression of pepper nsLTPs *CaLTP1* and *CaLTP2* in tobacco enhanced resistance to the oomycete pathogen *Phytophthora nicotianae* and the bacterial pathogen *Pseudomonas syringae pv tabaci* [19]. Similarly, overexpression of *StLTP10* in potato resulted in increased resistance to *P. infestans* [20].

nsLTPs are also involved in responses to abiotic stresses. T-DNA mutants of the Arabidopsis *Disease-Related Nonspecific Lipid Transfer Protein 1* (*DRNA1*) gene have decreased tolerance to salt stress [21]. Likewise, a T-DNA mutant of the *Lipid Transfer Protein 3* (*LTP3*) gene has increased sensibility to freezing, drought, and oxidative stress [22]. Conversely, overexpression of *LTP3* enhanced tolerance to freezing and drought [22]. Likewise, overexpression of the Arabidopsis *Azelaic Acid-Induced 1* (*AZI1*) nsLTP gene in Arabidopsis [23], the *Oryza sativa Drought-Induced LTP* (*OsDIL*) gene in rice [24], the pepper *CaLTP1* gene in Arabidopsis [25], and the *NtLTP4* gene in tobacco and potato [26,27] enhanced tolerance to drought and salt stresses.

In a previous study, we investigated by RNA-seq the molecular response of potato (*S. tuberosum*) to *Potato Virus Y* (PVY) inoculation [28]. PVY is one of the most devastating pathogens in potato production worldwide [29]. We identified several genes, including *StnsLTPI.33,* whose expression was strongly repressed in response to PVY inoculation (Goyer et al., 2015). Here, we investigated the role of *StnsLTPI.33*, which encodes a putative nsLTP, and showed that the overexpression of *StnsLTPI.33* increases susceptibility to *A. solani* and decreases tolerance to salt stress.

## 2. Results

### 2.1. The Gene PGSC0003DMG400031236 Encodes a Putative nsLTP

**Figure 1:**
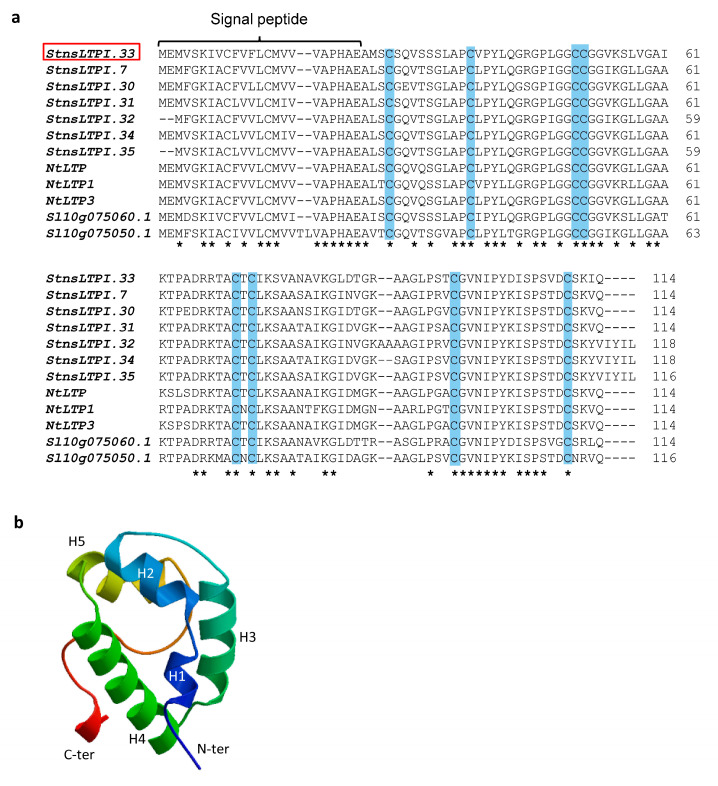
Protein sequence alignment and 3D model of StnsLTPI.33. (**a**) Amino acid sequence alignment of *StnsLTPI.33* from the variety Premier Russet with another type I nsLTP from potato (St), tobacco (Nt), and tomato (Sl). Asterisks indicate conserved amino acids. Cysteines from the eight cysteines motif are highlighted in blue. (**b**) Three-dimensional model of *StnsLTPI.33* generated by SWISS-PRO.

We previously reported that the expression of the gene *PGSC0003DMG400031236* (*Soltu.DM.10G018670.1*) in the potato variety Premier Russet was downregulated in response to PVY [28]. This gene is annotated as an nsLTP in Spud DB (http://spuddb.uga.edu/index.shtml [accessed on 1 June 2023]). We subsequently cloned and sequenced its cDNA, which encodes a 114-amino-acid-long protein (Figure 1a) that contains the 8CM C-Xn-C-Xn-CC-Xn-CXC-Xn-C-Xn-C, which is characteristic of plant nsLTPs. Based on the spacing pattern between cysteines, Li et al. [30] classified the protein encoded by this gene as a type I nsLTP, a group of 36 members in potato, and named it StnsLTPI.33. We used this denomination in this paper henceforth. A scan of the StnsLTPI.33 amino acid sequence in InterPro showed that the protein contains the Pfam domain PF00234 that is characteristic of LTPs and associated the protein with the InterPro GO terms ‘lipid transport’ (GO:0006869) and ‘lipid binding’ (GO:0008289). The SignalP5.0 software predicts an N-terminal 24-amino-acid-long signal peptide (Figure 1a) with a likelihood score of a secretory pathway localization of 0.9993. The mature protein has a predicted molecular weight of 9.1 kDa and an isoelectric point of 8.9. Further, the protein structure homology-modelling server SWISS-MODEL predicts a protein that comprises five α-helices (Figure 1b).

### 2.2. Gene Expression in Different Plant Tissues and in Response to Hormones

**Figure 2:**
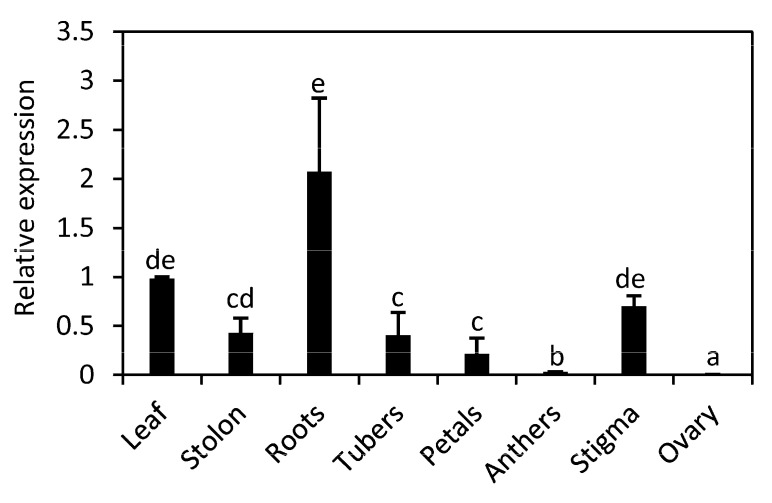
Relative *StnsLTPI.33* transcript levels in various organs of potato. Organs were collected from the potato variety Premier Russet. Expression levels were determined by qPCR. Data were normalized with the housekeeping genes *18S rRNA* and *L2*. Data are means ± SE of at least three biological replicates. Identical letters indicate that there was no significant difference between organs as determined by ANOVA (*p* < 0.05).

We analyzed the expression of *StnsLTPI.33* by qPCR in different organs in the variety Premier Russet (Figure 2). *StnsLTPI.33* transcripts were at the highest relative levels in the roots and the leaves. They were also detected in stolon, tubers, petals, and stigma, but were at low levels in anthers and ovary.

**Figure 3:**
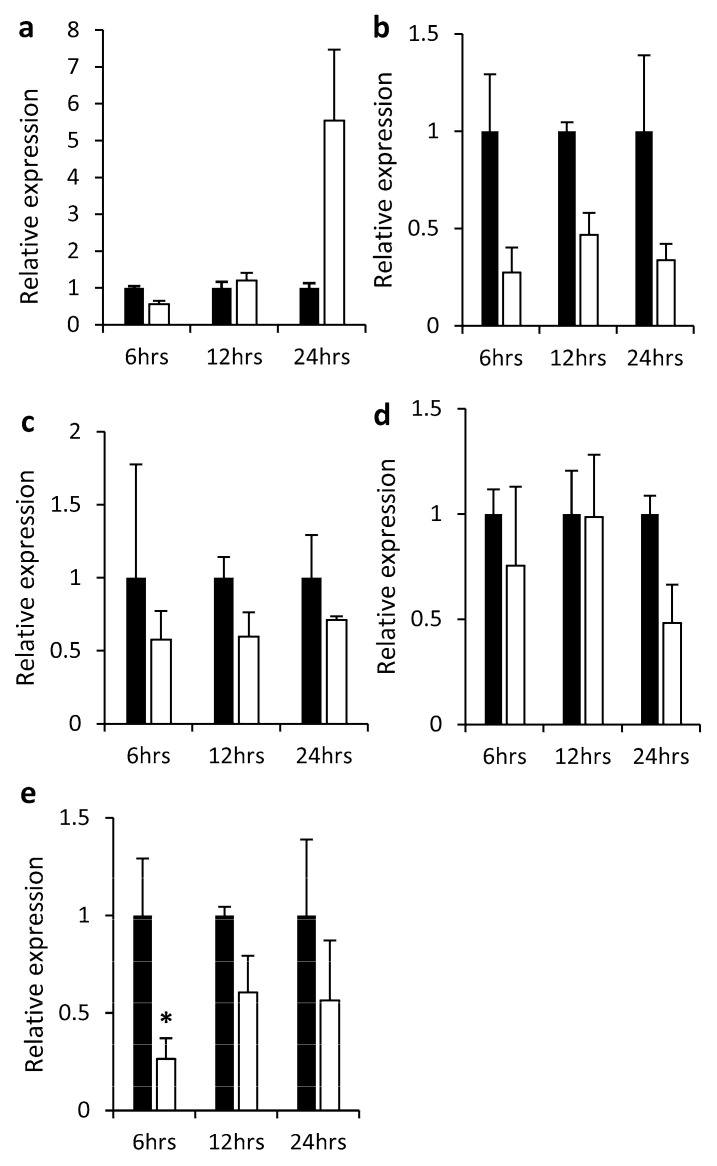
Relative expression of *StnsLTPI.33* gene in response to hormones. (**a**) Methyl jasmonate (Me-JA). (**b**) Aminocyclopropanecarboxylic acid (ACC). (**c**) Salicylic acid (SA). (**d**) Abscisic acid (ABA). (**e**) Gibberellic acid (GA). Plants grown in a greenhouse were treated with 1 mM ACC, 1 mM gibberellic acid (GA), 1 mM methyl jasmonate (Me-JA), 1 mM abscisic acid (ABA), or 1 mM salicylic acid (SA). For the Me-JA treatment, three plants were sprayed with a total of 100 mL of hormone solution or water as control until run-off and kept in covered translucent boxes. For the ACC, ABA, SA, and GA treatments, we harvested three leaves from each of the three plants for each treatment and dipped them in 50 mL of the hormone solution or water control in square Petri dishes. Samples were collected at 6, 12, and 24 h after treatment and frozen in liquid nitrogen until analysis by qPCR. Data were normalized with the housekeeping genes *18S rRNA* and *L2*. Data are means ± SE of at least three biological replicates. Asterisks indicate that there was a significant difference between treatments as determined by the Student’s *t*-test (*p* < 0.05). Black bars indicate the relative expression level in mock control samples normalized to 1, while white bars indicate the relative expression level in samples treated with hormones.

To obtain more information on the functions of *StnsLTPI.33,* we searched for *cis*-regulatory elements in the 1000 bp promoter region of *StnsLTPI.33* using PlantCARE. We found *cis*-acting regulatory elements that are responsive to auxin and methyl jasmonate (Me-JA) (Table S1). Me-JA is a precursor of jasmonic acid, an important player in plant defense responses. This suggests that *StnsLTPI.33* might be responsive to Me-JA treatment. Consistent with this hypothesis, *StnsLTPI.33* transcripts accumulated over five-fold 24 h after Me-JA treatment (Figure 3a), although the difference with the mock was not statistically significant. We also checked the expression of *StnsLTPI.33* in response to the other hormones salicylic acid (SA), aminocyclopropanecarboxylic acid (ACC), the precursor of ethylene, abscisic acid (ABA), and gibberellic acid (GA). All these hormones reduced the expression of the *StnsLTPI.33* at the time points tested (Figure 3b–e), although the differences between treated and control samples were not statistically significant, except for GA 6 h after treatment (Figure 3e). It is noteworthy that *StnsLTPI.33* is a low-expressed gene in general, and that changes in its expression are somewhat difficult to detect even with a sensitive method like qPCR.

### 2.3. Subcellular Localization of StnsLTPI.33

**Figure 4:**
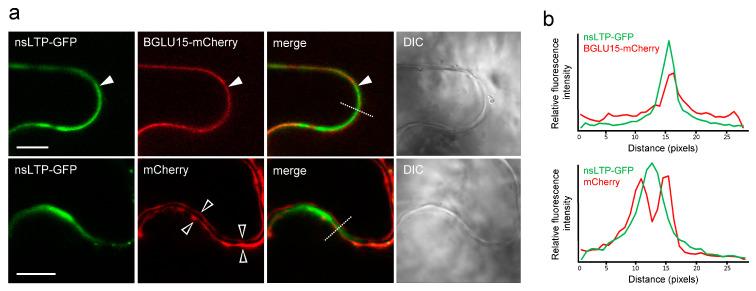
Localization of StnsLTPI.33 to the apoplast in *N. benthamiana* leaf cells. (**a**) Representative confocal laser-scanning microscope (CLSM) micrographs of portions of *Agrobacterium*-infiltrated leaf epidermal cells co-transformed (as indicated by panel labels) with nsLTP-GFP and either BGLU15-mCherry, serving as an apoplastic marker protein [32], or mCherry alone, serving as a cytosolic marker protein. Shown also are the corresponding merged and differential interference (DIC) images. Solid arrowheads in the top row indicate examples of the colocalization of nsLTP-GFP and BGLU15-mCherry in the apoplast/cell wall; open arrowheads in the bottom row indicate the localization of mCherry to the cytosol in the two neighboring leaf epidermal cells, with nsLTP-GFP localized in the shared apoplast/cell wall. Stippled lines in the merged images represent the regions of the cells that correspond to the fluorescence intensity profiles shown in the graphs in (**b**). Note in (**b**) that the fluorescence intensity profiles attributable to nsLTP-GFP and BLU15-mCherry mostly overlap, while those for nsLTP-GFP and mCherry are more distinct. Scale bars in (**a**) = 10 µm.

As mentioned above, the *StnsLTPI.33*-encoded protein is predicted to be localized in the secretory pathway and, thus, possibly the apoplast. To verify this, we cloned the full coding sequence of *StnsLTPI.33* upstream of the green fluorescent protein (nsLTP-GFP). We then used *Agrobacterium* to transiently express nsLTP-GFP in *N. benthamiana* leaves [31]. To identify subcellular compartments, we co-expressed nsLTP-GFP with either mCherry alone, which served as cytosolic marker protein, or BGLU15-mCherry, which served as an apoplastic marker protein [32]. As shown in Figure 4, nsLTP-GFP was adjacent to, but did not colocalize with, mCherry in the cytosol (Figure 4). By contrast, nsLTP-GFP consistently colocalized with the apoplastic marker BGLU15-mCherry (Figure 4), indicating that StnsLTPI.33 is localized in the apoplast.

### 2.4. Phenotypic Characterization of Transgenic Lines Overexpressing StnsLTPI.33

**Figure 5:**
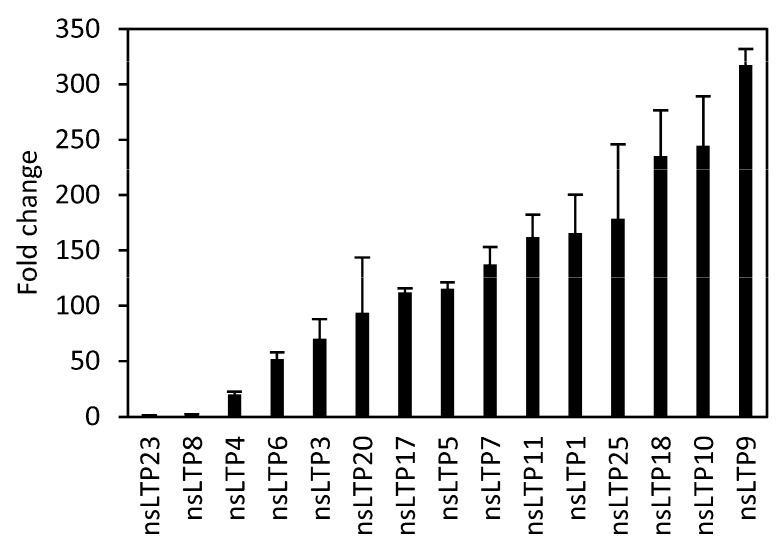
*StnsLTPI.33* transcript levels in transgenic potato lines expressing the coding sequence of *StnsLTPI.33* under the control of the CaMV35S promoter. Plantlets were grown on MS medium in magenta boxes. Leaf samples were used for RNA extraction and qPCR. Data were normalized with the housekeeping genes *18S rRNA* and *L2*. Data are means ± SE of three biological replicates.

Next, we generated plants that express the *StnsLTPI.33* cDNA under the CaMV35S promoter by stable transformation with *A. tumefaciens*. We obtained 15 independent transgenic plants and used qPCR to determine the expression levels of *StnsLTPI.33* in leaf tissues of plants grown in magenta boxes (Figure 5). The expression of *StnsLTPI.33* increased from ~1.2 to ~317 times that of the control. Based on these results, we selected two independent lines with different levels of expression for further characterization: nsLTP10, which expressed the transgene at the highest level (nsLTP9 died and could not be used), and nsLTP11, which showed a mid-range expression level of the transgene.

**Figure 6:**
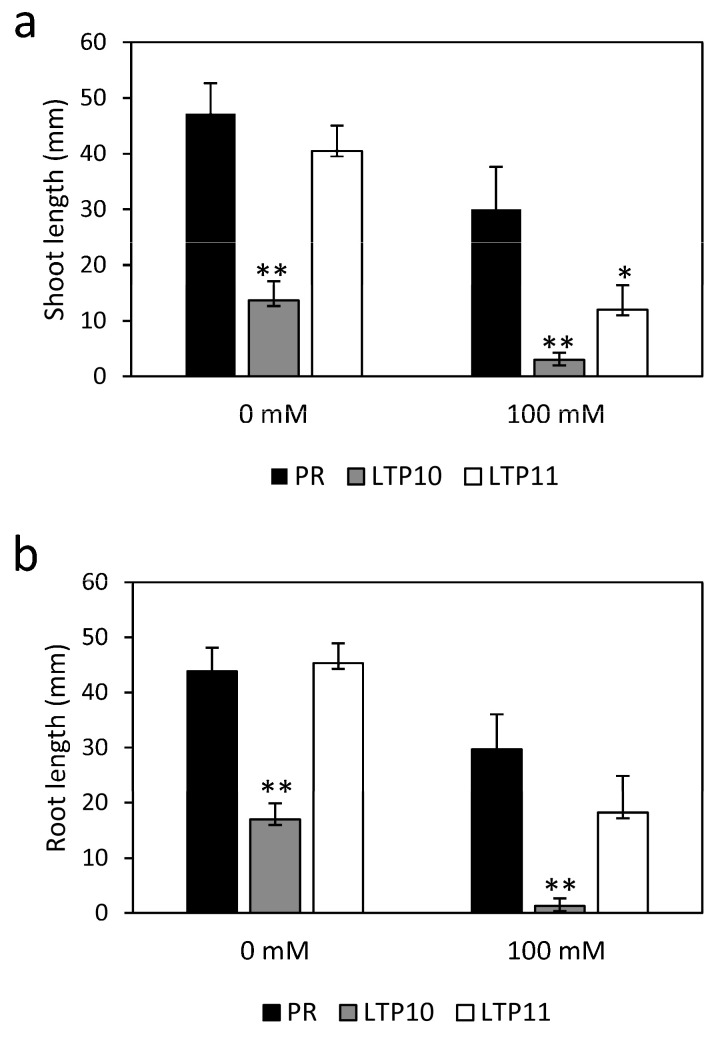
Shoot (**a**) and root (**b**) lengths of *StnsLTPI.33*-overexpressing lines grown on MS medium or MS medium with 100 mM NaCl. Data are means ± SE of six biological replicates. Asterisks indicate that there was a significant difference between *StnsLTPI.33*-overexpressing lines and the control Premier Russet (PR), as determined by the Student’ *t*-test. *, *p* < 0.1; **, *p* < 0.05.

Line nsLTP10 grew smaller than the control in tissue culture (smaller shoots and roots), while nsLTP11 was not statistically different from the control (Figure 6a,b and Figure S1). These differences between the two overexpression lines might be due to the different levels of expression of the transgene in these two lines, although we did not check the corresponding protein levels. Under greenhouse conditions, the smaller phenotype of *StnsLTPI.33*-overexpression lines was still visible but was attenuated (Figure S2).

### 2.5. Response of StnsLTPI.33-Overexpression Lines to Salt Stress

**Figure 7:**
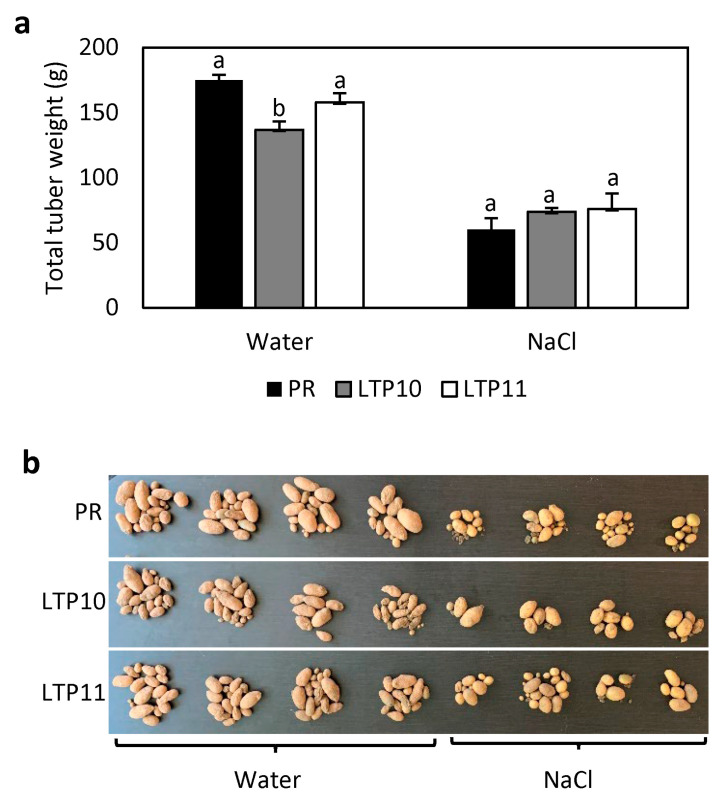
Tuber yield of *StnsLTPI.33*-overexpressing lines nsLTP10 and nsLTP11 compared to the control Premier Russet (PR). (**a**) Total weight of harvested tubers. (**b**) Pictures of harvested tubers. Identical letters indicate that there was no significant difference between lines as determined by ANOVA (*p* < 0.05).

Because plant nsLTPs have been involved in the response to abiotic stress [22,26,33,34], we checked whether *StnsLTPI.33* was involved in plants’ response to abiotic stress. For this, we grew *StnsLTPI.33*-overexpressing lines on MS medium containing 100 mM NaCl. The short shoots and roots phenotype of the *StnsLTPI.33*-overexpressing line nsLTP10 grown on MS medium was further exacerbated when plants were grown on MS medium with 100 mM NaCl compared to the control Premier Russet (Figure 6a,b). Shoots of line nsLTP11 were also significantly shorter than those of the control under salt stress conditions. Length of shoots of lines nsLTP10 and nsLTP11 were 10% and 40%, respectively, of that of the control Premier Russet when grown on MS with 100 mM NaCl, compared to 29% and 85% when grown on MS medium. Likewise, the length of the roots of lines nsLTP10 and nsLTP11 were 4% and 61%, respectively, of that of the control Premier Russet when grown on MS with 100 mM NaCl, compared to 39% and 103% when grown on MS medium. We also grew plants in pots in a greenhouse and irrigated them with water or a 100 mM NaCl solution. Under water irrigation, the tuber yield of line nsLTP10 was slightly affected, while that of nsLTP11 was not (Figure 7). Under salt irrigation, the yield of all genotypes decreased evenly, and there was no difference between *StnsLTPI.33*-overexpressing lines and control plants. These results show that the overexpression of *StnsLTPI.33* further exacerbated the growth phenotype when plants were grown in tissue culture but had little effect on tuber yield when plants were grown in a greenhouse and whether plants were irrigated with water or salt.

### 2.6. Response of StnsLTPI.33-Overexpression Lines to PVY

Because *StnsLTPI.33* was one of the most responsive genes to PVY inoculation [28], we tested the response of the *StnsLTPI.33*-overexpressing lines compared to the control Premier Russet to three different strains of PVY (PVY^O^, PVY^NTN^, and PVY^N-Wilga^). Premier Russet contains a strain-specific *Ny* resistance gene that confers resistance against PVY^O^ and PVY^N-Wilga^, while it is susceptible to PVY^NTN^ [35]. Thus, we might expect decreased resistance to PVY^O^ and PVY^N-Wilga^ if *StnsLTPI.33* is a negative regulator of resistance, or increased resistance to PVY^NTN^ if *StnsLTPI.33* is a positive regulator of resistance. To test this, we inoculated leaflets mechanically and evaluated the systemic spread of the virus in non-inoculated leaves (i.e., in-season infection) and in progeny tubers (i.e., seed-borne infection) by RT-PCR. We repeated the experiment three times (Table 1).

In general, leaf and tuber infection rates of the control Premier Russet with PVY^O^ and PVY^N-Wilga^ were low, which is consistent with the presence of a strain-specific *Ny* resistance gene in Premier Russet against PVY^O^ and PVY^N-Wilga^, while infection rates with PVY^NTN^ were high. However, infection rates with PVY^O^, PVY^N-Wilga^, and PVY^NTN^ in *StnsLTPI.33*-overexpressing lines were similar to those in Premier Russet. These results suggest that *StnsLTPI.33* does not play a major role in the response to PVY.

### 2.7. Response of StnsLTPI.33-Overexpression Lines to Alternaria Solani

**Figure 8:**
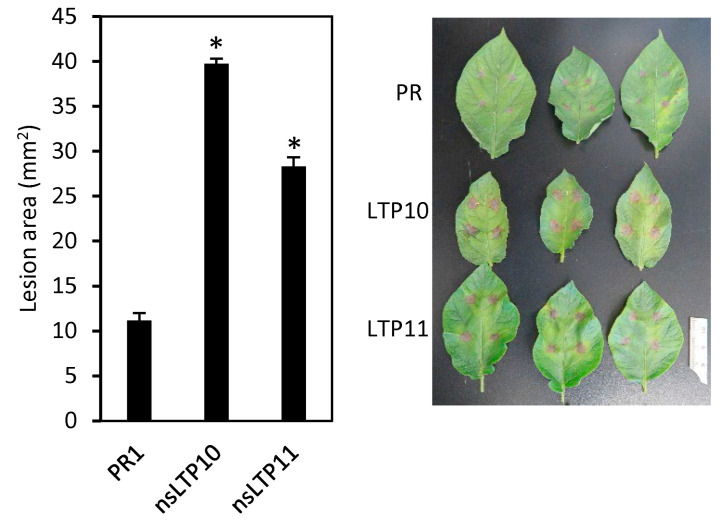
Disease response to *Alternaria solani* in *StnsLTPI.33*-overexpressing lines nsLTP10 and nsLTP11. Asterisks indicate that there was a significant difference between *StnsLTPI.33*-overexpressing lines and the control Premier Russet (PR) as determined by the Student’s *t*-test (*p* < 0.05).

Several studies have shown an association between nsLTPs and plant defense response against fungal, bacterial, and oomycete pathogens [19,20,26,36]. This prompted us to evaluate the response of *StnsLTPI.33*-overexpressing lines against the fungal pathogen *A. solani*. Necrotic lesions caused by *A. solani* were significantly larger on leaves of *StnsLTPI.33*-overexpressing lines than on those of the Premier Russet control (Figure 8), showing that *StnsLTPI.33*-overexpressing lines were more susceptible to *A. solani*.

### 2.8. StnsLTPI.33-Overexpression Lines Accumulate Superoxide Anions

**Figure 9:**
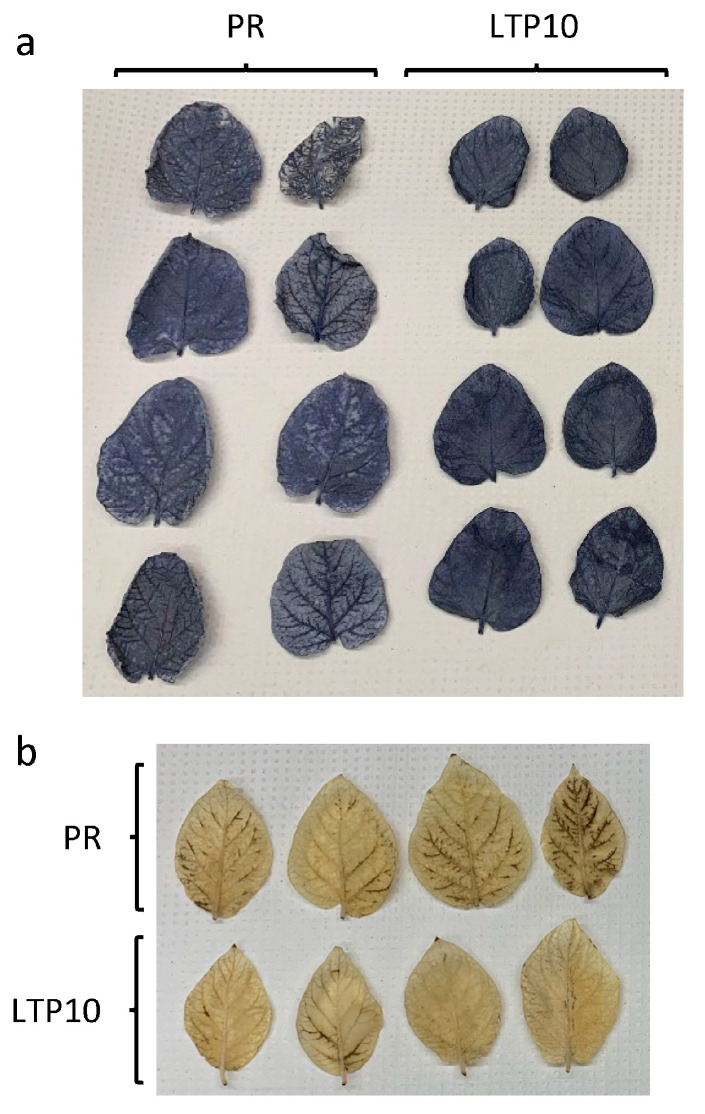
NBT (**a**) and DAB (**b**) staining of leaflets of the *StnsLTPI.33*-overexpressing line nsLTP10.

To investigate the molecular changes in *StnsLTPI.33*-overexpressing lines that might explain the smaller size phenotype and the higher susceptibility to *A. solani*, we stained leaves of the line nsLTP10 with Nitro Blue Tetrazolium (NBT) and 3,3′-diaminobenzidine (DAB). NBT is an indicator of the level of superoxide anions, while DAB is an indicator of the level of hydrogen peroxide. We observed a heavier blue coloration in all leaf tissues of nsLTP10 compared to the control Premier Russet (Figure 9), indicating an overaccumulation of superoxide anions in this line. Conversely, staining of leaves with DAB showed a slightly lighter coloration in veins of nsLTP10 compared to the control Premier Russet, indicating a lower accumulation of hydrogen peroxide in vascular tissues of this line.

**Figure 10:**
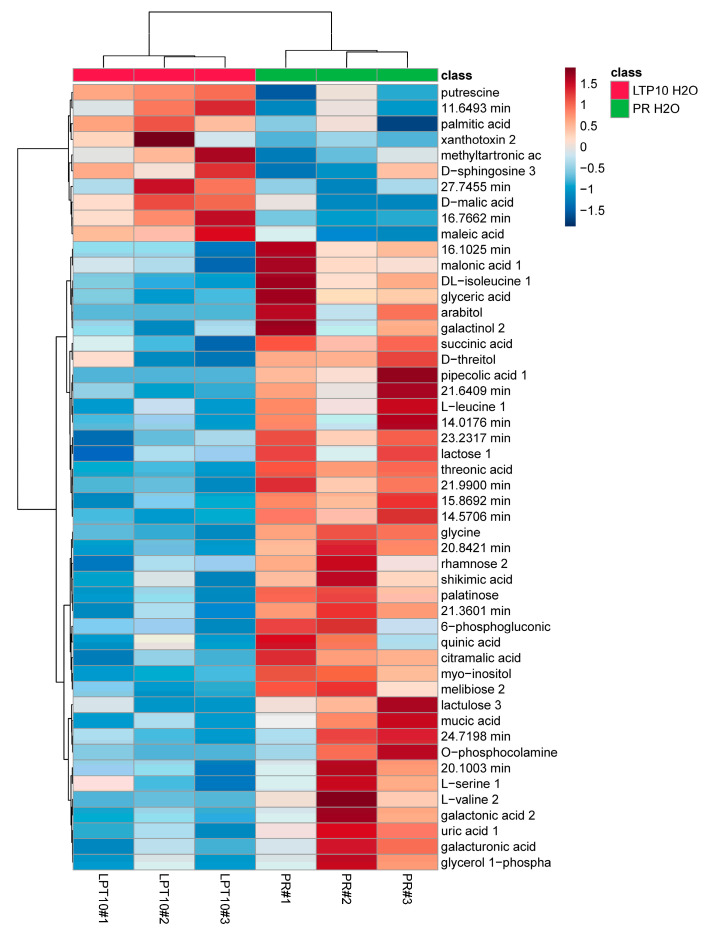
Heatmap of the top 50 leaf metabolites in the *StnsLTPI.33*-overexpressing line LTP10 and the control Premier Russet. The top 50 differential metabolites between samples based on ANOVA are shown. Samples and metabolites were clustered based on Ward’s linkage.

We also used GC-MS to examine general metabolic changes in the *StnsLTPI.33*-overexpressing line nsLTP10 and the Premier Russet control. A heatmap analysis of the top 50 leaf metabolites shows that samples were grouped by genotype (Figure 10), indicating good reproducibility between biological replicates. In general, more metabolites were lower in abundance in nsLTP10 compared to the control Premier Russet (Figure 10 and Tables S2 and S3). By restricting our analysis to metabolites whose changes were significantly different at a *p*-value < 0.05, fifteen metabolites accumulated at different levels in nsLTP10 compared to Premier Russet (Table S3). Among these, only one, putrescine, accumulated in larger amounts in nsLTP10 compared to Premier Russet. Pipecolic acid was the metabolite found in the lowest relative amount in nsLTP10 compared to Premier Russet, followed by threonic acid and glycine. Other metabolites that accumulated differentially included sugars, sugar alcohols, organic acids, and amino acids. Two compounds with retention times 20.84 min and 15.86 min were also found in lower amounts in nsLTP10 compared to Premier Russet, but they could not be identified by our methods.

## 3. Discussion

In this study, we investigated the role of *StnsLTPI.33*, one member of the large nsLTP gene family in potato. This family member first caught our interest because it is one of the most strongly differentially expressed genes in response to PVY inoculation in potato [28]. Several studies have previously reported the involvement of plant nsLTPs in virus resistance. Pepper *CaLTP1* expression was induced by an incompatible strain of tobacco mosaic virus [37], and suppression of its expression enhanced pepper plants’ susceptibility to mosaic mottle virus [19]. The effect of its constitutive overexpression on virus resistance was not reported [19]. Potato *StLTP6* expression was induced by PVY and potato virus S, and overexpression of *StLTP6* promoted accumulation of viral mRNAs, while silencing it reduced viral mRNAs accumulation [38]. In the case of *StnsLTPI.33*, we observed a decrease of its transcripts in an incompatible interaction with PVY [28]. Therefore, we hypothesized that *StnsLTPI.33* might also play a role in the response to a viral pathogen and expected changes in infection rates in *StnsLTPI.33*-overexpression plants. However, our data show that constitutive overexpression of *StnsLTPI.33* had no effect on the infection rate of plants inoculated with either compatible or incompatible strains of PVY, suggesting that this gene may not play a major role in susceptibility or resistance to PVY. Another possibility is that changes in susceptibility or resistance to PVY can only be observed if the StnsLTPI.33 protein levels reach a certain threshold. Therefore, it would be interesting to verify the accumulation level of the StnsLTPI.33 protein in *StnsLTPI.33*-overexpressing plants.

Most studies have investigated the function of plant nsLTPs in the context of non-viral pathogens and have reported that overexpression of *nsLTP* genes enhances resistance to fungal, bacterial, and oomycete pathogens [19,20,25,39,40,41,42,43]. Surprisingly, our work shows that plants that constitutively overexpress *StnsLTPI.33* are more susceptible to *A. solani*, indicating that *StnsLTPI.33* is a susceptibility gene in the response of potato plants to this fungal pathogen. The accumulation of superoxide anions in *StnsLTPI.33*-overexpressing plants might explain the higher susceptibility of these plants to *A. solani*. Indeed, a burst of reactive oxygen species increased susceptibility to the early blight disease caused by *A. solani* in potato [44]. Conversely, this burst conferred resistance to the late blight pathogen *P. infestans* [44]. Therefore, it would be interesting to test the resistance of *StnsLTPI.33*-overexpressing plants to other pathogens to determine whether *StnsLTPI.33* is a susceptibility gene specific to *A. solani*. If it is, then hyperaccumulation of superoxide anions likely accelerated cell death and benefited the necrotrophic lifestyle of *A. solani*. Previous studies have shown that necrotrophic pathogens often highjack defense signaling to promote the accumulation of reactive oxygen species and accelerate cell death to their benefit [44,45,46]. If *StnsLTPI.33* is a susceptibility gene to a broader range of pathogens, then the molecular mechanism of susceptibility might be related to other factors, pipecolic acid being a potential candidate. Indeed, the level of this non-protein amino acid was more than eight times lower in the *StnsLTPI.33*-overexpressing plant nsLTP10 compared to the control Premier Russet, indicating that *StnsLTPI.33* negatively regulates the levels of pipecolic acid, directly or indirectly. Pipecolic acid is essential in the activation of local resistance and systemic acquired resistance to bacterial and fungal pathogens [47,48,49,50].

*StnsLTPI.33*-overexpressing plants also had a shorter shoot and root phenotype, in particular when plants were grown in tissue culture. LTPs are associated with plant growth, although the exact molecular mechanism remains unclear. It could be through cell wall loosening and/or lipid deposition [5,10,51]. However, this function of LTPs in cell expansion does not explain the shorter phenotype of *StnsLTPI.33*-overexpressing plants. Hence, it is more likely that *StnsLTPI.33* has an indirect association with plant growth via metabolic changes triggered by its overexpression. For instance, reactive oxygen species are important signaling molecules for plant growth and development [52]. An imbalance of reactive oxygen species such as that seen in *StnsLTPI.33*-overexpressing plants likely has an effect on the normal growth and development of these plants. That StnsLTPI.33 is localized in the apoplast where superoxide anions are produced by NADPH oxidases [53,54] reinforces the idea of a relationship between StnsLTPI.33 and the production of reactive oxygen species. Putrescine was another metabolite whose content significantly changed in *StnsLTPI.33*-overexpressing plants. It accumulated 2.7-fold in the nsLTP10 line compared to the control. Putrescine is one of the main polyamines found in plants [55]. Polyamines play important roles in plant growth and development and oxidative stress. Whether putrescine plays a role in the growth phenotype of *StnsLTPI.33*-overexpressing plants remains unclear.

In conclusion, this work shows that StnsLTPI.33 is an apoplastic protein whose gene is associated with plant growth and susceptibility to *A. solani*, and likely plays a role in the production of superoxide anions. It illustrates a new role for nsLTPs. However, future investigations that include StnsLTPI.33 protein quantification are needed to better understand the molecular mechanisms involved.

## 4. Materials and Methods

### 4.1. Phylogenetic Analysis

Amino acid sequences of nsLTPs from potato (*S. tuberosum*), tobacco (*Nicotiana tabacum*), tomato (*S. lycopersicon*), and *Arabidopsis thaliana* were aligned using Muscle version 3.8.31 [56] using default settings for multiple pairwise alignments. Potato nsLTP amino acid sequences were retrieved from SpudDB (http://solanaceae.plantbiology.msu.edu/index.shtml, [accessed on 24 August 2020]). Tobacco and tomato nsLTP amino acid sequences were obtained from the Solanaceae Genomics Network (https://solgenomics.net/, [accessed on 24 August 2020]).

### 4.2. Plant Growth Conditions

Potato plantlets variety Premier Russet were propagated on Murashige and Skoog (MS) medium (4.41 g L^−1^ MS modified BC potato salts, 3% sucrose, 100 mg L^−1^ myo-inositol, 2 mg L^−1^ glycine, 0.5 mg L^−1^ nicotinic acid, 0.5 mg L^−1^ pyridoxine, 0.1 mg L^−1^ thiamin, 20 µg L^−1^ 1-naphthaleneacetic acid, 0.5 g L^−1^ 2-(N-morpholino)ethanesulfonic acid, 8 g L^−1^ phytoagar, pH 5.6) under a 10 h photoperiod at 22 °C and 36% relative humidity.

For salt stress assays in tissue culture, we made new cuttings from four-week-old plantlets and placed them in magenta boxes containing 50 mL MS agar medium with 0 or 100 mM NaCl. In these experiments, we omitted myo-inositol from the medium. Each magenta box contained six plantlets (one genotype per box per treatment). After 17 days under the growth conditions above, we gently uprooted each plantlet to measure shoot and root length. This experiment was repeated three times.

All other experiments with potato plants were conducted in a greenhouse. For this, three-week-old plantlets from tissue culture were transferred into soil (four parts potting mix, one part sand) with slow-release fertilizer (Osmocote Plus) in 4 L pots in a greenhouse. Greenhouse temperature conditions were set at 21 °C at day and 15 °C at night, and supplemental daylight was provided with 400 W high-pressure sodium lamps to maintain a 14 h photoperiod. For the salt stress assay, each genotype was grown in three to four replicates per treatment (salt or control) in a randomized complete block design. We watered plants every three to five days with 500 mL (drench) of a 100-mM NaCl solution or water for controls for one month starting five weeks after transfer to soil. We harvested tubers 20 days after the end of the salt treatment. *N. benthamiana* plants used in subcellular localization experiments were grown in soil with a 16 h/8 h day/night cycle at 22 °C and a light intensity of 50 µmol m^−2^ s^−1^.

### 4.3. Generation of StnsLTPI.33-Overexpressing Potato Plants

Total RNAs were extracted from leaves from the potato variety Premier Russet using the hot phenol method as previously described [57] and treated with DNase (Ambion^®^ DNA-free™ kit, LifeTechnologies, Carlsbad, CA, USA). cDNAs were synthesized by M-MuLV Reverse Transcriptase (New England Biolabs, Ipswich, MA, USA) using an oligo(dT)_18_ primer, and the PGSC0003DMG400031236 (*StnsLTPI.33*)-encoded cDNA was amplified using PrimeSTAR Max DNA Polymerase (Takara Bio USA, San Jose, CA, USA) using the nsLTPfwd and nsLTPrev primers (Table S4). The 587-bp amplicon was directly cloned into pCR^TM^4Blunt TOPO^®^ vector (ThermoFisher Scientific, Waltham, MA, USA), and the resulting construct was introduced into One Shot TOP10 *E. coli* cells (ThermoFisher Scientific). Eighteen kanamycin-resistant isolated colonies were then cultured in LB medium supplemented with 50 mg L^−1^ kanamycin. Plasmid DNA was extracted from each culture and sent for Sanger sequencing. Sequences alignment showed that *PGSC0003DMG400031236* has four identical alleles in Premier Russet (one clone, 3b, had a 60 bp insertion that likely corresponds to an unspliced variant) (Figure S3). Clone 4-1 was used as a template to amplify a 361 bp amplicon using the nsLTPattFwd and nsLTPattRev primers (Table S4). The 361 bp amplicon was then ligated into pDONR^TM^/Zeo vector (ThermoFisher Scientific) using BP clonase following the manufacturer’s recommendations and then subcloned into the pMDC32 plant binary vector [58] by recombination using LR clonase. The final construct was verified by restriction digestion and Sanger sequencing. The DNA construct was introduced into the potato variety Premier Russet by *A. tumefaciens* (strain EHA105)-mediated stable transformation as previously described [59].

### 4.4. Quantitative Reverse Transcription PCR

Total RNAs were isolated as described above. cDNAs were synthesized from 1 µg RNA using M-MuLV reverse Transcriptase (New England Biolabs). RT-qPCR analysis was performed on an Mx3000P real-time PCR detection system (Stratagene, San Diego, CA, USA) using Brilliant III Ultra-Fast SYBR^®^ Green QPCR Master Mix (Agilent, Santa Clara, CA, USA). Potato *L2*, *18S rRNA,* and *EF1-α* genes were used as housekeeping genes for normalization. Three biological replicates sampled from three individual plants were used for each treatment, and two technical replicates for each biological replicate were assayed by qRT-qPCR. Expression levels were calculated by the 2^-∆∆Ct^ method as described by Ref. [60]. Primer sets used for gene expression analysis are listed in Table S4.

### 4.5. Hormone Treatments

Plants grown in a greenhouse were treated with 1 mM ACC, 1 mM GA, 1 mM ABA, 1 mM Me-JA, or 1 mM SA. For Me-JA treatment, three plants were sprayed with a total of 100 mL of hormone solution or water as control until run-off and kept in covered translucent boxes. For the ACC, ABA, SA, and GA treatments, we harvested three leaves from each of the three plants for each treatment and dipped them in 50 mL of the hormone solution or water control in square petri dishes. Samples were collected at 6, 12, and 24 h after treatment and frozen in liquid nitrogen until analysis.

### 4.6. Subcellular Localization

Leaves of ~4-week-old *N. benthamiana* plants were transiently (co) transformed by infiltration with *A. tumefaciens* (strain LBA4404) carrying the selected binary vector (s). Detailed procedures for *A. tumefaciens* growth, transformation, infiltration, and processing of *N. benthamiana* leaf material for microscopy have been described previously [61,62].

To construct pMDC83/nsLTP-GFP (containing the two copies of the constitutive *35S* Cauliflower Mosaic Virus promoter (CaMV35S) and encoding StnsLTPI.33 appended at its C terminus to the GFP), the open reading frame (ORF) of *StnsLTPI.33* with *attB1* and *attB2* sequences in 5′ and 3′, respectively, was synthesized by GeneScript (Table S4) and cloned in the entry vector pDONR^TM^/Zeo using BP clonase. The cloned plasmid was transformed into *E. coli* and confirmed by restriction enzyme digestion. We then performed LR recombination reaction with the positive entry clone and the pMDC83 vector [58] to generate 2xCaMV35S::nsLTP::GFP expression clone. The expression clone was confirmed by restriction enzyme digestion and sequencing at the University of Guelph Genomics Facility.

To construct pMDC32/BGLU15-mCherry (containing the CaMV35S promoter and encoding Arabidopsis β-glucosidase 15 [AT2G44450] appended at its C terminus to the monomeric red fluorescent protein mCherry), the ORF of the BGLU15-mCherry fusion was amplified (via polymerase chain reaction [PCR]) using pRTL2/BGLU15-Cherry [32] as template and the primers BGLU15-mcherry pDONR Fp and BGLU15-mcherry pDONR Rp (Table S4). Oligonucleotide primers were custom synthesized by Sigma-Aldrich (St. Louis, MO, USA). Thereafter, the BGLU15-mCherry ORF was subcloned in pDONR^TM^/Zeo and then in the plant binary expression vector pMDC32/ChC [63] using Gateway cloning technology [58]. The sequence of pMDC32/BGLU15-mCherry plasmid was verified using automated DNA sequencing performed at the University of Guelph Genomics Facility. The construction of pMDC32/mCherry, encoding mCherry alone and serving in this study as a cytosolic marker protein, has been described elsewhere [64].

Imaging of transformed *N. benthamiana* leaves was carried out three days after infiltrations using a Leica SP5 CLSM equipped with a 63x glycerol-immersion objective (NA = 1.3) and five laser systems, including an argon-ion laser; green, orange, and red helium-neon lasers; and a Radius 405 nm laser (Leica Microsystems, Wetzlar, Germany). All images of leaf cells were acquired as single optical sections (i.e., z-sections) and saved as 512- × 512-pixel digital images. Excitations and emission signals for fluorescent fusion proteins (GFP 488/500-530 nm for GFP and 543/565-585 nm for mCherry) were collected sequentially as single optical sections in double-labeling experiments as described previously [65]. Single-labelling experiments showed no detectable crossover at the settings used for data collection. Micrographs of plant cells shown in individual figures are representative of at least three separate experiments, including > 20 transformed *N. benthamiana* leaf cells. Fluorescence intensity profiles of selected areas (i.e., lines) of micrographs were generated using the “Plot Profile” tool in ImageJ (v.1.43; https://imagej.nih.gov/ij, [accessed on 1 July 2022]) [66].

### 4.7. Detection of Hydrogen Peroxide and Superoxide Anion Radicals

We used DAB (Sigma-Aldrich) and NBT (Sigma-Aldrich) for staining analysis of hydrogen peroxide and superoxide anion radicals, respectively, using the methods described by Li et al. [67].

### 4.8. Metabolite Analysis by GC-MS

Leaf metabolites were analyzed by GC-MS (Agilent 7890B GC, Agilent 5977B MSD). Metabolites were extracted from 50 mg of leaf tissue in a solution of water:methanol:chloroform (1:2.5:1). Ribitol was added to each sample as an internal standard. Samples were placed on ice for 5 min on a shaking platform rotating at 130 rpm and then centrifuged at 4 °C for 2 min at 21,000× *g* to pellet cellular debris. The supernatant was transferred to a clean microcentrifuge tube and 280 µL of water was added to separate the aqueous phase from the organic phase. After a 2 min centrifugation at 21,000× *g*, the upper aqueous phase was collected and placed into a clean microcentrifuge tube. The samples were frozen at −80 °C, then placed into a centrifugal vacuum concentrator and lyophilized to dryness overnight. Dried samples were stored at −80 °C until further analysis. A no-tissue extraction control (i.e., reagent blank) was included to assess if detected peaks are plant tissue-specific.

For GC-MS analysis, the dry samples were derivatized in a two-step process. First, 40 µL of 20 mg mL^−1^ methoxamine HCl (CovaChem, Loves Park, IL, USA) dissolved in pyridine (Sigma-Aldrich) was added to each sample, and the samples were incubated at 37 °C for 90 min in a 1800 rpm shaking incubator. Second, 80 µL of N-methyl-N-trimethylsilyl-trifluoroacetamide with 1% chlorotrimethylsilane (CovaChem) was added to each sample, and the samples were incubated at 37 °C for 30 min in a shaking incubator at 1800 rpm. The derivatized samples were then transferred to autosampler vials, and one µL of each sample was injected with a 15:1 split into an Agilent 7890B GC system with a 30 m + 10 m Duraguard × 0.25 mm × 0.25 µm DB-5MS + DG Agilent column. The oven temperature was kept at 60 °C for 1 min, then ramped to 300 °C at a rate of 10 °C/min and held at 300 °C for 10 min. Analytes were detected with an Agilent 5977B MSD in EI mode scanning from 50 to 600 m/z. The injection order of the samples was randomized. The no-tissue extraction control was injected after every three injections of tissue sample to control for potential carry over from previous samples.

We used AMDIS [68] for mass spectrum analysis, component identification, and peak area quantification, and the Agilent Fiehn 2013 GC/MS Metabolomics RTL Library [69] for automated component identification. Matches to both retention time and mass spectrum were required for a positive identification, with AMDIS settings of a minimum match factor of 70, a retention time window of 0.2 min, strong match factor penalties, and maximum penalty of 60. Components identified in the no-tissue extraction controls were removed from the data set.

To determine relative abundance, peak areas of components were first normalized to the respective measured fresh weight of extracted tissue. The normalized peak areas were then normalized to the peak area of the internal standard ribitol. Statistical analyses were performed with Metaboanalyst 5.0 (https://www.metaboanalyst.ca/ [accessed on 21 June 2023]) using the following settings: ‘replace missing values with 1/5 limit of detection’ and ‘normalization by reference feature ribitol’.

### 4.9. PVY Strains, Inoculation and Detection

Fresh tobacco (*N. tabaccum* var. Samsun *NN*) leaves inoculated with PVY^O^ (no accession number, isolated from Aberdeen, Idaho, in 1999), PVY^NTN^ (Genebank ID FJ204166), PVY^N-wilga^ (Genebank ID HQ912863), or buffer (mock) were used as source of viral inoculum. Leaves of potato plants were inoculated as previously described [70]. The inoculation was repeated after seven days to ensure efficient viral inoculation. Potato plants were arranged in a randomized split-block design with six replications per treatment. Plants were inoculated three-to-four weeks after transfer from tissue culture to soil. To detect virus translocation to the tubers, three tubers were harvested from each plant, treated with 7 ppm gibberellic acid 3 (GA_3_) for three weeks at 27 °C to break dormancy. Tubers were then planted in 4 L pots with potting mix, and the grow outs were tested for the presence of PVY. We detected PVY by reverse transcription (RT)-PCR as previously described [35].

### 4.10. Alternaria Solani Disease Assays

*A. solani* was grown on V8 agar medium (10% clarified V8 juice, 1.5% CaCO_3_, and 12.7% agar) under continuous light. Spores were harvested after 10 days by flooding the plates with 10 mL sterile water and gently spreading it over the mycelium to dislodge the conidia using plastic hockey spreaders. The spore solution was filtered through four layers of cheesecloth to separate the spores from the mycelium. Spores were counted using a Neubauer chamber, and concentration was adjusted to 15,000–30,000 spores mL^−1^.

Potato plants were used four weeks after transfer to soil in all the assays. Four detached leaflets per plant from each of four plants per genotype were harvested and arranged in germination trays. The leaves were drop inoculated on the adaxial side with 10 µL of the spore inoculum and covered with clear humidity domes to maintain high humidity. Lesion area was measured at four days after inoculation using ImageJ [66].

### 4.11. Statistical Analysis of the Results

Data were analyzed for statistical significance using Student *t*-test or Analysis of Variance (ANOVA) in Excel or Vassarstats. For metabolite analysis, we used MetaboAnalyst 5.0 [71].

## Figures and Tables

**Table 1:** Rate of in-season and seed-borne infection of Premier Russet (PR) and lines overexpressing *StnsLTPI.33* (LTP10 and 11) in three independent experiments.

		Experiment 1		Experiment 2		Experiment 3	
Treatment	Potato Line	In-Season ^a^	Seedborne ^b^	In-Season ^a^	Seedborne ^b^	In-Season ^a^	Seedborne ^b^
Mock	PR	0/6	0/6	0/6	0/6	0/6	0/5
	LTP10	0/6	0/5	1/6	0/5	0/3	0/2
	LTP11	0/5	0/5	0/5	0/5	0/5	0/4
PVY^O^	PR	0/6	0/6	0/6	0/6	1/6	0/6
	LTP10	0/6	0/6	0/6	0/4	1/4	0/2
	LTP11	0/5	0/5	0/5	0/5	2/5	0/5
PVY^N-Wilga^	PR	0/6	0/6	4/6	3/6	2/6	0/6
	LTP10	0/4	0/4	2/5	4/5	0/4	0/4
	LTP11	0/6	0/5	0/6	4/6	1/6	0/5
PVY^NTN^	PR	4/6	4/6	5/6	5/6	5/6	5/6
	LTP10	5/6	5/6	5/6	5/5	4/5	2/3
	LTP11	4/6	4/6	4/4	4/4	6/6	5/6

^a^ Plants were mechanically inoculated with PVY on foliage (i.e., in-season infection). PVY detection was performed by RT-PCR on non-inoculated leaves. ^b^ Tubers were harvested, planted, and then tested for PVY infection by RT-PCR (i.e., seedborne infection) on foliage.

## Data Availability

All the data generated and analyzed in this study are included in this published article.

## Supplementary Materials

The following supporting information can be downloaded at: https://www.mdpi.com/article/10.3390/plants12173129/s1, Figure S1: Phenotype of *StnsLTPI.33*-overexpressing lines nsLTP10 and nsLTP11 grown on MS media (pictures on the left with blue tape) or MS media containing 100 mM NaCl (pictures on the right with green tape) in magenta boxes; Figure S2: Phenotype of *StnsLTPI.33*-overexpressing lines nsLTP10 and nsLTP11 grown in pots in a greenhouse. The picture on the top comprises the control Premier Russet and the nsLTP10 line. The picture at the bottom comprises the control Premier Russet and the nsLTP11 line; Figure S3: Nucleotide sequence alignment of 18 cloned *PGSC0003DMG400031236* cDNAs. The PGSC0003DMG400031236-encoded cDNA was amplified by PCR, cloned in pCR^TM^4Blunt TOPO^®^ vector (ThermoFisher Scientific), and the resulting construct was introduced into One Shot TOP10 *E. coli* cells (ThermoFisher Scientific). Eighteen kanamycin-resistant isolated colonies were then cultured in LB medium supplemented with 50 mg/L kanamycin. Plasmid DNA was extracted from each culture and sent for Sanger sequencing. Sequences alignment showed that *PGSC0003DMG400031236* has four identical alleles in Premier Russet (one clone, 3b, had a 60 bp insertion that likely corresponds to an unspliced variant); Table S1: The putative *cis*-acting regulatory elements in the promoter of the *StnsLTPI.33* gene; Table S2: Amounts of leaf metabolites in the StnslTPI.33-overexpression line nsLTP10 and the control Premier Russet (PR). Values were normalized to both sample weight and internal standard. Prominent peaks in the chromatograms that were not identified by the automated search process are labelled with their retention time; Table S3: List of differentially accumulated metabolites in the *StnslTPI.33*-overexpression line LTP10 compared to the control Premier Russet (PR). This list was generated by Volcano plot analysis in MetaboAnalyst 5.0 with the following settings: FC > 2, raw *p*-value < 0.1, missing values: unchecked “Remove features with > 50% missing values”, checked “Replace by LoDs (1/5 of the minimum positive value of each variable)”; Table S4: Primers and sequences used in this study.
