# Supplementary material for: Potato Non-Specific Lipid Transfer Protein StnsLTPI.33 Is Associated with the Production of Reactive Oxygen Species, Plant Growth, and Susceptibility to Alternaria solani
Source: Plants (Basel). 2023 Aug 31;12(17):3129. doi: 10.3390/plants12173129 (PMC10490331; doi:10.3390/plants12173129)
Supplement: Supplementary file 1 [file plants-12-03129-s001.zip › plants-2564693-supplementary.pdf]

Supplementary Materials

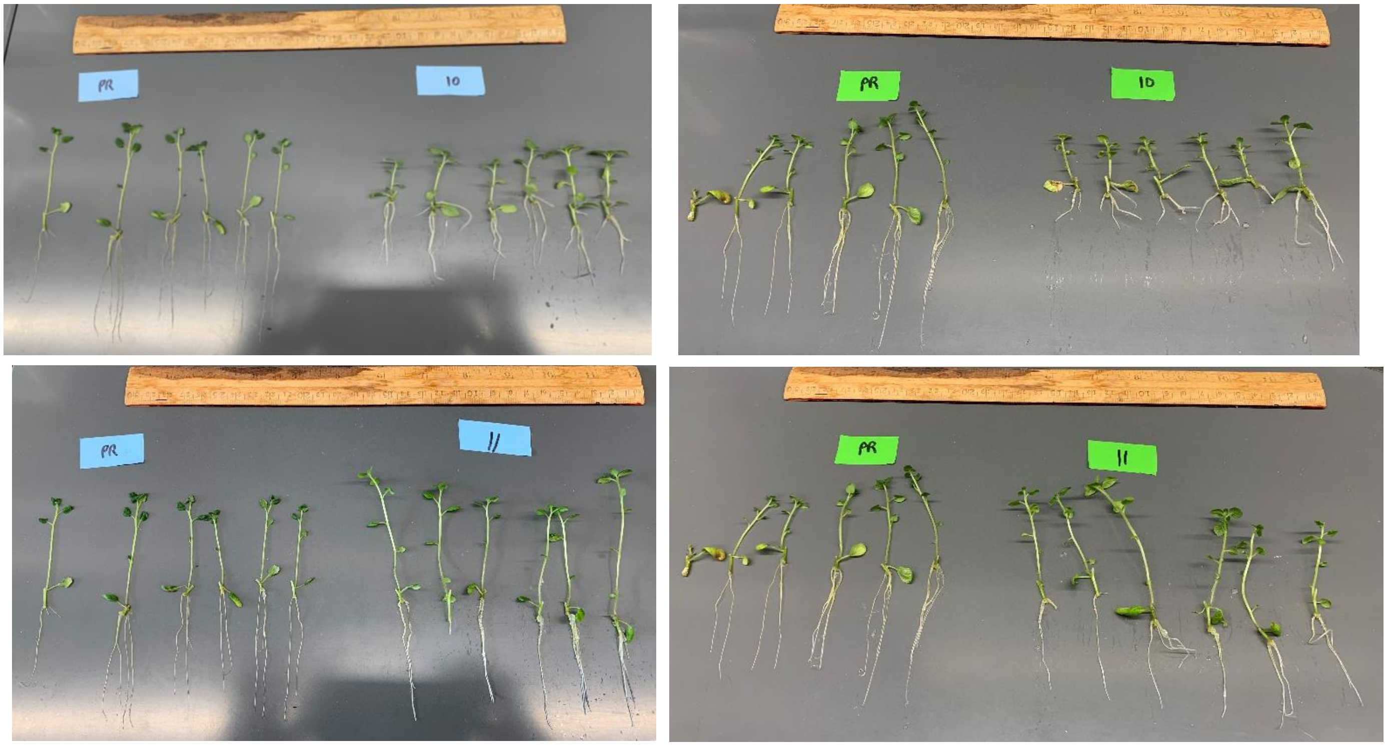

Figure S1: Phenotype of *StnsLTPI.33*-overexpressing lines nsLTP10 and nsLTP11 grown on MS media (pictures on the left with blue tape) or MS media containing 100 mM NaCl (pictures on the right with green tape) in Magenta boxes.

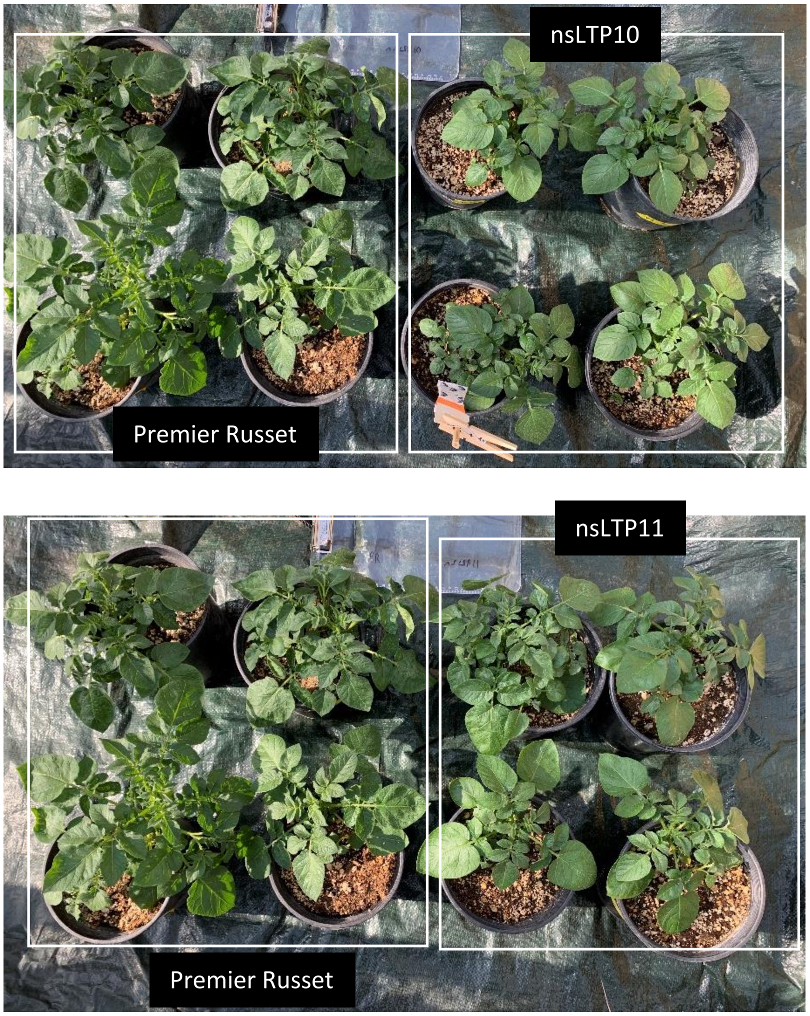

Figure S2: Phenotype of *StnsLTPI.33*-overexpressing lines nsLTP10 and nsLTP11 grown in pots in a greenhouse. The picture on the top comprises the control Premier Russet and the nsLTP10 line. The picture at the bottom comprises the control Premier Russet and the nsLTP11 line.

```
          1       10        20        30        40        50        60        70        80        90       100       110       120       130
          |--------+---------+---------+---------+---------+---------+---------+---------+---------+---------+---------+---------+---------|
  clone4-1 CTCAAGCAAATACATTACATTACATTTAATATAAACATGGAAATGGTTAGCAAAATTGTATGCTTTGTGTTTTTGTGCATGGTGGTGGTTGCACCCCATGCAGAGGCAATGAGCTGTAGCCAAGTTTCGT
  clone4-2 CTCAAGCAAATACATTACATTACATTTAATATAAACATGGAAATGGTTAGCAAAATTGTATGCTTTGTGTTTTTGTGCATGGTGGTGGTTGCACCCCATGCAGAGGCAATGAGCTGTAGCCAAGTTTCGT
 clone4-16 CTCAAGCAAATACATTACATTACATTTAATATAAACATGGAAATGGTTAGCAAAATTGTATGCTTTGTGTTTTTGTGCATGGTGGTGGTTGCACCCCATGCAGAGGCAATGAGCTGTAGCCAAGTTTCGT
 clone4-15 CTCAAGCAAATACATTACATTACATTTAATATAAACATGGAAATGGTTAGCAAAATTGTATGCTTTGTGTTTTTGTGCATGGTGGTGGTTGCACCCCATGCAGAGGCAATGAGCTGTAGCCAAGTTTCGT
 clone4-14 CTCAAGCAAATACATTACATTACATTTAATATAAACATGGAAATGGTTAGCAAAATTGTATGCTTTGTGTTTTTGTGCATGGTGGTGGTTGCACCCCATGCAGAGGCAATGAGCTGTAGCCAAGTTTCGT
 clone4-13 CTCAAGCAAATACATTACATTACATTTAATATAAACATGGAAATGGTTAGCAAAATTGTATGCTTTGTGTTTTTGTGCATGGTGGTGGTTGCACCCCATGCAGAGGCAATGAGCTGTAGCCAAGTTTCGT
 clone4-12 CTCAAGCAAATACATTACATTACATTTAATATAAACATGGAAATGGTTAGCAAAATTGTATGCTTTGTGTTTTTGTGCATGGTGGTGGTTGCACCCCATGCAGAGGCAATGAGCTGTAGCCAAGTTTCGT
 clone4-11 CTCAAGCAAATACATTACATTACATTTAATATAAACATGGAAATGGTTAGCAAAATTGTATGCTTTGTGTTTTTGTGCATGGTGGTGGTTGCACCCCATGCAGAGGCAATGAGCTGTAGCCAAGTTTCGT
 clone4-10 CTCAAGCAAATACATTACATTACATTTAATATAAACATGGAAATGGTTAGCAAAATTGTATGCTTTGTGTTTTTGTGCATGGTGGTGGTTGCACCCCATGCAGAGGCAATGAGCTGTAGCCAAGTTTCGT
  clone4-9 CTCAAGCAAATACATTACATTACATTTAATATAAACATGGAAATGGTTAGCAAAATTGTATGCTTTGTGTTTTTGTGCATGGTGGTGGTTGCACCCCATGCAGAGGCAATGAGCTGTAGCCAAGTTTCGT
  clone4-8 CTCAAGCAAATACATTACATTACATTTAATATAAACATGGAAATGGTTAGCAAAATTGTATGCTTTGTGTTTTTGTGCATGGTGGTGGTTGCACCCCATGCAGAGGCAATGAGCTGTAGCCAAGTTTCGT
  clone4-6 CTCAAGCAAATACATTACATTACATTTAATATAAACATGGAAATGGTTAGCAAAATTGTATGCTTTGTGTTTTTGTGCATGGTGGTGGTTGCACCCCATGCAGAGGCAATGAGCTGTAGCCAAGTTTCGT
 clone4-4b CTCAAGCAAATACATTACATTACATTTAATATAAACATGGAAATGGTTAGCAAAATTGTATGCTTTGTGTTTTTGTGCATGGTGGTGGTTGCACCCCATGCAGAGGCAATGAGCTGTAGCCAAGTTTCGT
  clone4-3 CTCAAGCAAATACATTACATTACATTTAATATAAACATGGAAATGGTTAGCAAAATTGTATGCTTTGTGTTTTTGTGCATGGTGGTGGTTGCACCCCATGCAGAGGCAATGAGCTGTAGCCAAGTTTCGT
 clone4-2b CTCAAGCAAATACATTACATTACATTTAATATAAACATGGAAATGGTTAGCAAAATTGTATGCTTTGTGTTTTTGTGCATGGTGGTGGTTGCACCCCATGCAGAGGCAATGAGCTGTAGCCAAGTTTCGT
 clone4-1b CTCAAGCAAATACATTACATTACATTTAATATAAACATGGAAATGGTTAGCAAAATTGTATGCTTTGTGTTTTTGTGCATGGTGGTGGTTGCACCCCATGCAGAGGCAATGAGCTGTAGCCAAGTTTCGT
  clone4-5 CTCAAGCAAATACATTACATTACATTTAATATAAACATGGAAATGGTTAGCAAAATTGTATGCTTTGTGTTTTTGTGCATGGTGGTGGTTGCACCCCATGCAGAGGCAATGAGCTGTAGCCAAGTTTCGT
 clone4-3b CTCAAGCAAATACATTACATTACATTTAATATAAACATGGAAATGGTTAGCAAAATTGTATGCTTTGTGTTTTTGTGCATGGTGGTGGTTGCACCCCATGCAGAGGCAATGAGCTGTAGCCAAGTTTCGT
 Consensus CTCAAGCAAATACATTACATTACATTTAATATAAACATGGAAATGGTTAGCAAAATTGTATGCTTTGTGTTTTTGTGCATGGTGGTGGTTGCACCCCATGCAGAGGCAATGAGCTGTAGCCAAGTTTCGT

          131    140       150       160       170       180       190       200       210       220       230       240       250       260
          |--------+---------+---------+---------+---------+---------+---------+---------+---------+---------+---------+---------+---------|
  clone4-1 CTAGCTTGGCTCCTTGTGTCCCTTATCTTCAAGGGCGCGGCCCTCTAGGAGGTTGTTGCGGTGGTGTTAAGAGTCTGGTGGGTGCAGTCAAGACCCCAGCGGACCGAAGGACAGCATGCACTTGTATAAA
  clone4-2 CTAGCTTGGCTCCTTGTGTCCCTTATCTTCAAGGGCGCGGCCCTCTAGGAGGTTGTTGCGGTGGTGTTAAGAGTCTGGTGGGTGCAGTCAAGACCCCAGCGGACCGAAGGACAGCATGCACTTGTATAAA
 clone4-16 CTAGCTTGGCTCCTTGTGTCCCTTATCTTCAAGGGCGCGGCCCTCTAGGAGGTTGTTGCGGTGGTGTTAAGAGTCTGGTGGGTGCAGTCAAGACCCCAGCGGACCGAAGGACAGCATGCACTTGTATAAA
 clone4-15 CTAGCTTGGCTCCTTGTGTCCCTTATCTTCAAGGGCGCGGCCCTCTAGGAGGTTGTTGCGGTGGTGTTAAGAGTCTGGTGGGTGCAGTCAAGACCCCAGCGGACCGAAGGACAGCATGCACTTGTATAAA
 clone4-14 CTAGCTTGGCTCCTTGTGTCCCTTATCTTCAAGGGCGCGGCCCTCTAGGAGGTTGTTGCGGTGGTGTTAAGAGTCTGGTGGGTGCAGTCAAGACCCCAGCGGACCGAAGGACAGCATGCACTTGTATAAA
 clone4-13 CTAGCTTGGCTCCTTGTGTCCCTTATCTTCAAGGGCGCGGCCCTCTAGGAGGTTGTTGCGGTGGTGTTAAGAGTCTGGTGGGTGCAGTCAAGACCCCAGCGGACCGAAGGACAGCATGCACTTGTATAAA
 clone4-12 CTAGCTTGGCTCCTTGTGTCCCTTATCTTCAAGGGCGCGGCCCTCTAGGAGGTTGTTGCGGTGGTGTTAAGAGTCTGGTGGGTGCAGTCAAGACCCCAGCGGACCGAAGGACAGCATGCACTTGTATAAA
 clone4-11 CTAGCTTGGCTCCTTGTGTCCCTTATCTTCAAGGGCGCGGCCCTCTAGGAGGTTGTTGCGGTGGTGTTAAGAGTCTGGTGGGTGCAGTCAAGACCCCAGCGGACCGAAGGACAGCATGCACTTGTATAAA
 clone4-10 CTAGCTTGGCTCCTTGTGTCCCTTATCTTCAAGGGCGCGGCCCTCTAGGAGGTTGTTGCGGTGGTGTTAAGAGTCTGGTGGGTGCAGTCAAGACCCCAGCGGACCGAAGGACAGCATGCACTTGTATAAA
  clone4-9 CTAGCTTGGCTCCTTGTGTCCCTTATCTTCAAGGGCGCGGCCCTCTAGGAGGTTGTTGCGGTGGTGTTAAGAGTCTGGTGGGTGCAGTCAAGACCCCAGCGGACCGAAGGACAGCATGCACTTGTATAAA
  clone4-8 CTAGCTTGGCTCCTTGTGTCCCTTATCTTCAAGGGCGCGGCCCTCTAGGAGGTTGTTGCGGTGGTGTTAAGAGTCTGGTGGGTGCAGTCAAGACCCCAGCGGACCGAAGGACAGCATGCACTTGTATAAA
  clone4-6 CTAGCTTGGCTCCTTGTGTCCCTTATCTTCAAGGGCGCGGCCCTCTAGGAGGTTGTTGCGGTGGTGTTAAGAGTCTGGTGGGTGCAGTCAAGACCCCAGCGGACCGAAGGACAGCATGCACTTGTATAAA
 clone4-4b CTAGCTTGGCTCCTTGTGTCCCTTATCTTCAAGGGCGCGGCCCTCTAGGAGGTTGTTGCGGTGGTGTTAAGAGTCTGGTGGGTGCAGTCAAGACCCCAGCGGACCGAAGGACAGCATGCACTTGTATAAA
  clone4-3 CTAGCTTGGCTCCTTGTGTCCCTTATCTTCAAGGGCGCGGCCCTCTAGGAGGTTGTTGCGGTGGTGTTAAGAGTCTGGTGGGTGCAGTCAAGACCCCAGCGGACCGAAGGACAGCATGCACTTGTATAAA
 clone4-2b CTAGCTTGGCTCCTTGTGTCCCTTATCTTCAAGGGCGCGGCCCTCTAGGAGGTTGTTGCGGTGGTGTTAAGAGTCTGGTGGGTGCAGTCAAGACCCCAGCGGACCGAAGGACAGCATGCACTTGTATAAA
 clone4-1b CTAGCTTGGCTCCTTGTGTCCCTTATCTTCAAGGGCGCGGCCCTCTAGGAGGTTGTTGCGGTGGTGTTAAGAGTCTGGTGGGTGCAGTCAAGACCCCAGCGGACCGAAGGACAGCATGCACTTGTATAAA
  clone4-5 CTAGCTTGGCTCCTTGTGTCCCTTATCTTCAAGGGCGCGGCCCTCTAGGAGGTTGTTGCGGTGGTGTTAAGAGTCTGGTGGGTGCAGTCAAGACCCCAGCGGACCGAAGGACAGCATGCACTTGTATAAA
 clone4-3b CTAGCTTGGCTCCTTGTGTCCCTTATCTTCAAGGGCGCGGCCCTCTAGGAGGTTGTTGCGGTGGTGTTAAGAGTCTGGTGGGTGCAGTCAAGACCCCAGCGGACCGAAGGACAGCATGCACTTGTATAAA
 Consensus CTAGCTTGGCTCCTTGTGTCCCTTATCTTCAAGGGCGCGGCCCTCTAGGAGGTTGTTGCGGTGGTGTTAAGAGTCTGGTGGGTGCAGTCAAGACCCCAGCGGACCGAAGGACAGCATGCACTTGTATAAA

          261    270       280       290       300       310       320       330       340       350       360       370       380       390
          |--------+---------+---------+---------+---------+---------+---------+---------+---------+---------+---------+---------+---------|
  clone4-1 ATCAGTAGCTAATGCTGTTAAGGGACTTGATACCGGCAGAGCCGCTGGTCTCCCTAGTACTTGTGGCGTTAACATCCCTTACGATATCAGCCCCTCCGTTGATTGCTCCAAG------------------
  clone4-2 ATCAGTAGCTAATGCTGTTAAGGGACTTGATACCGGCAGAGCCGCTGGTCTCCCTAGTACTTGTGGCGTTAACATCCCTTACGATATCAGCCCCTCCGTTGATTGCTCCAAG------------------
 clone4-16 ATCAGTAGCTAATGCTGTTAAGGGACTTGATACCGGCAGAGCCGCTGGTCTCCCTAGTACTTGTGGCGTTAACATCCCTTACGATATCAGCCCCTCCGTTGATTGCTCCAAG------------------
 clone4-15 ATCAGTAGCTAATGCTGTTAAGGGACTTGATACCGGCAGAGCCGCTGGTCTCCCTAGTACTTGTGGCGTTAACATCCCTTACGATATCAGCCCCTCCGTTGATTGCTCCAAG------------------
 clone4-14 ATCAGTAGCTAATGCTGTTAAGGGACTTGATACCGGCAGAGCCGCTGGTCTCCCTAGTACTTGTGGCGTTAACATCCCTTACGATATCAGCCCCTCCGTTGATTGCTCCAAG------------------
 clone4-13 ATCAGTAGCTAATGCTGTTAAGGGACTTGATACCGGCAGAGCCGCTGGTCTCCCTAGTACTTGTGGCGTTAACATCCCTTACGATATCAGCCCCTCCGTTGATTGCTCCAAG------------------
 clone4-12 ATCAGTAGCTAATGCTGTTAAGGGACTTGATACCGGCAGAGCCGCTGGTCTCCCTAGTACTTGTGGCGTTAACATCCCTTACGATATCAGCCCCTCCGTTGATTGCTCCAAG------------------
 clone4-11 ATCAGTAGCTAATGCTGTTAAGGGACTTGATACCGGCAGAGCCGCTGGTCTCCCTAGTACTTGTGGCGTTAACATCCCTTACGATATCAGCCCCTCCGTTGATTGCTCCAAG------------------
 clone4-10 ATCAGTAGCTAATGCTGTTAAGGGACTTGATACCGGCAGAGCCGCTGGTCTCCCTAGTACTTGTGGCGTTAACATCCCTTACGATATCAGCCCCTCCGTTGATTGCTCCAAG------------------
  clone4-9 ATCAGTAGCTAATGCTGTTAAGGGACTTGATACCGGCAGAGCCGCTGGTCTCCCTAGTACTTGTGGCGTTAACATCCCTTACGATATCAGCCCCTCCGTTGATTGCTCCAAG------------------
  clone4-8 ATCAGTAGCTAATGCTGTTAAGGGACTTGATACCGGCAGAGCCGCTGGTCTCCCTAGTACTTGTGGCGTTAACATCCCTTACGATATCAGCCCCTCCGTTGATTGCTCCAAG------------------
  clone4-6 ATCAGTAGCTAATGCTGTTAAGGGACTTGATACCGGCAGAGCCGCTGGTCTCCCTAGTACTTGTGGCGTTAACATCCCTTACGATATCAGCCCCTCCGTTGATTGCTCCAAG------------------
 clone4-4b ATCAGTAGCTAATGCTGTTAAGGGACTTGATACCGGCAGAGCCGCTGGTCTCCCTAGTACTTGTGGCGTTAACATCCCTTACGATATCAGCCCCTCCGTTGATTGCTCCAAG------------------
  clone4-3 ATCAGTAGCTAATGCTGTTAAGGGACTTGATACCGGCAGAGCCGCTGGTCTCCCTAGTACTTGTGGCGTTAACATCCCTTACGATATCAGCCCCTCCGTTGATTGCTCCAAG------------------
 clone4-2b ATCAGTAGCTAATGCTGTTAAGGGACTTGATACCGGCAGAGCCGCTGGTCTCCCTAGTACTTGTGGCGTTAACATCCCTTACGATATCAGCCCCTCCGTTGATTGCTCCAAG------------------
 clone4-1b ATCAGTAGCTAATGCTGTTAAGGGACTTGATACCGGCAGAGCCGCTGGTCTCCCTAGTACTTGTGGCGTTAACATCCCTTACGATATCAGCCCCTCCGTTGATTGCTCCAAG------------------
  clone4-5 ATCAGTAGCTAATGCTGTTAAGGGACTTGATACCGGCAGAGCCGCTGGTCTCCCTAGTACTTGTGGCGTTAACATCCCTTACGATATCAGCCCCTCCGTTGATTGCTCCAAG------------------
 clone4-3b ATCAGTAGCTAATGCTGTTAAGGGACTTGATACCGGCAGAGCCGCTGGTCTCCCTAGTACTTGTGGCGTTAACATCCCTTACGATATCAGCCCCTCCGTTGATTGCTCCAAGTATATACGTTAATTTATA
 Consensus ATCAGTAGCTAATGCTGTTAAGGGACTTGATACCGGCAGAGCCGCTGGTCTCCCTAGTACTTGTGGCGTTAACATCCCTTACGATATCAGCCCCTCCGTTGATTGCTCCAAG

          391    400       410       420       430       440       450       460       470       480       490       500       510       520
          |--------+---------+---------+---------+---------+---------+---------+---------+---------+---------+---------+---------+---------|
  clone4-1 ------------------------------------------ATCCAGTAAGGTTGATGAAAGCTGATCCACTACACAATATGGAAGTCACTATATATTTTATTATTACGAGAAGAATAAAAGGGATCGA
  clone4-2 ------------------------------------------ATCCAGTAAGGTTGATGAAAGCTGATCCACTACACAATATGGAAGTCACTATATATTTTATTATTACGAGAAGAATAAAAGGGATCGA
 clone4-16 ------------------------------------------ATCCAGTAAGGTTGATGAAAGCTGATCCACTACACAATATGGAAGTCACTATATATTTTATTATTACGAGAAGAATAAAAGGGATCGA
 clone4-15 ------------------------------------------ATCCAGTAAGGTTGATGAAAGCTGATCCACTACACAATATGGAAGTCACTATATATTTTATTATTACGAGAAGAATAAAAGGGATCGA
 clone4-14 ------------------------------------------ATCCAGTAAGGTTGATGAAAGCTGATCCACTACACAATATGGAAGTCACTATATATTTTATTATTACGAGAAGAATAAAAGGGATCGA
 clone4-13 ------------------------------------------ATCCAGTAAGGTTGATGAAAGCTGATCCACTACACAATATGGAAGTCACTATATATTTTATTATTACGAGAAGAATAAAAGGGATCGA
 clone4-12 ------------------------------------------ATCCAGTAAGGTTGATGAAAGCTGATCCACTACACAATATGGAAGTCACTATATATTTTATTATTACGAGAAGAATAAAAGGGATCGA
 clone4-11 ------------------------------------------ATCCAGTAAGGTTGATGAAAGCTGATCCACTACACAATATGGAAGTCACTATATATTTTATTATTACGAGAAGAATAAAAGGGATCGA
 clone4-10 ------------------------------------------ATCCAGTAAGGTTGATGAAAGCTGATCCACTACACAATATGGAAGTCACTATATATTTTATTATTACGAGAAGAATAAAAGGGATCGA
  clone4-9 ------------------------------------------ATCCAGTAAGGTTGATGAAAGCTGATCCACTACACAATATGGAAGTCACTATATATTTTATTATTACGAGAAGAATAAAAGGGATCGA
  clone4-8 ------------------------------------------ATCCAGTAAGGTTGATGAAAGCTGATCCACTACACAATATGGAAGTCACTATATATTTTATTATTACGAGAAGAATAAAAGGGATCGA
  clone4-6 ------------------------------------------ATCCAGTAAGGTTGATGAAAGCTGATCCACTACACAATATGGAAGTCACTATATATTTTATTATTACGAGAAGAATAAAAGGGATCGA
 clone4-4b ------------------------------------------ATCCAGTAAGGTTGATGAAAGCTGATCCACTACACAATATGGAAGTCACTATATATTTTATTATTACGAGAAGAATAAAAGGGATCGA
  clone4-3 ------------------------------------------ATCCAGTAAGGTTGATGAAAGCTGATCCACTACACAATATGGAAGTCACTATATATTTTATTATTACGAGAAGAATAAAAGGGATCGA
 clone4-2b ------------------------------------------ATCCAGTAAGGTTGATGAAAGCTGATCCACTACACAATATGGAAGTCACTATATATTTTATTATTACGAGAAGAATAAAAGGGATCGA
 clone4-1b ------------------------------------------ATCCAGTAAGGTTGATGAAAGCTGATCCACTACACAATATGGAAGTCACTATATATTTTATTATTACGAGAAGAATAAAAGGGATCGA
  clone4-5 ------------------------------------------ATCCAGTAAGGTTGATGAAAGCTGATCCACTACACAATATGGAAGTCACTATATATTTTATTATTACGAGAAGAATAAAAGGGATCGA
 clone4-3b AGTTTATACTATATAGTAATTATTTTGTGATACTATATATAGATCCAGTAAGGTTGATGAAAGCTGATCCACTACACAATATGGAAGTCACTATATATTTTATTATTACGAGAAGAATAAAAGGGATCGA
 Consensus                                           ATCCAGTAAGGTTGATGAAAGCTGATCCACTACACAATATGGAAGTCACTATATATTTTATTATTACGAGAAGAATAAAAGGGATCGA

          521    530       540       550       560       570       580       590       600       610       620       630       640       650
          |--------+---------+---------+---------+---------+---------+---------+---------+---------+---------+---------+---------+---------|
  clone4-1 ACTAGTTTGATCCTGTTTTGTATTTTGTATCGATCTGTGTTTCTTTTTATTATTTAGTGTCTTTTAATTTGGAGTCATATATAGCTAGTCAGTGTCTTGTAATGAAACTTTGGTTGTTGCTTCTTCAACA
  clone4-2 ACTAGTTTGATCCTGTTTTGTATTTTGTATCGATCTGTGTTTCTTTTTATTATTTAGTGTCTTTTAATTTGGAGTCATATATAGCTAGTCAGTGTCTTGTAATGAAACTTTGGTTGTTGCTTCTTCAACA
 clone4-16 ACTAGTTTGATCCTGTTTTGTATTTTGTATCGATCTGTGTTTCTTTTTATTATTTAGTGTCTTTTAATTTGGAGTCATATATAGCTAGTCAGTGTCTTGTAATGAAACTTTGGTTGTTGCTTCTTCAACA
 clone4-15 ACTAGTTTGATCCTGTTTTGTATTTTGTATCGATCTGTGTTTCTTTTTATTATTTAGTGTCTTTTAATTTGGAGTCATATATAGCTAGTCAGTGTCTTGTAATGAAACTTTGGTTGTTGCTTCTTCAACA
 clone4-14 ACTAGTTTGATCCTGTTTTGTATTTTGTATCGATCTGTGTTTCTTTTTATTATTTAGTGTCTTTTAATTTGGAGTCATATATAGCTAGTCAGTGTCTTGTAATGAAACTTTGGTTGTTGCTTCTTCAACA
 clone4-13 ACTAGTTTGATCCTGTTTTGTATTTTGTATCGATCTGTGTTTCTTTTTATTATTTAGTGTCTTTTAATTTGGAGTCATATATAGCTAGTCAGTGTCTTGTAATGAAACTTTGGTTGTTGCTTCTTCAACA
 clone4-12 ACTAGTTTGATCCTGTTTTGTATTTTGTATCGATCTGTGTTTCTTTTTATTATTTAGTGTCTTTTAATTTGGAGTCATATATAGCTAGTCAGTGTCTTGTAATGAAACTTTGGTTGTTGCTTCTTCAACA
 clone4-11 ACTAGTTTGATCCTGTTTTGTATTTTGTATCGATCTGTGTTTCTTTTTATTATTTAGTGTCTTTTAATTTGGAGTCATATATAGCTAGTCAGTGTCTTGTAATGAAACTTTGGTTGTTGCTTCTTCAACA
 clone4-10 ACTAGTTTGATCCTGTTTTGTATTTTGTATCGATCTGTGTTTCTTTTTATTATTTAGTGTCTTTTAATTTGGAGTCATATATAGCTAGTCAGTGTCTTGTAATGAAACTTTGGTTGTTGCTTCTTCAACA
  clone4-9 ACTAGTTTGATCCTGTTTTGTATTTTGTATCGATCTGTGTTTCTTTTTATTATTTAGTGTCTTTTAATTTGGAGTCATATATAGCTAGTCAGTGTCTTGTAATGAAACTTTGGTTGTTGCTTCTTCAACA
  clone4-8 ACTAGTTTGATCCTGTTTTGTATTTTGTATCGATCTGTGTTTCTTTTTATTATTTAGTGTCTTTTAATTTGGAGTCATATATAGCTAGTCAGTGTCTTGTAATGAAACTTTGGTTGTTGCTTCTTCAACA
  clone4-6 ACTAGTTTGATCCTGTTTTGTATTTTGTATCGATCTGTGTTTCTTTTTATTATTTAGTGTCTTTTAATTTGGAGTCATATATAGCTAGTCAGTGTCTTGTAATGAAACTTTGGTTGTTGCTTCTTCAACA
 clone4-4b ACTAGTTTGATCCTGTTTTGTATTTTGTATCGATCTGTGTTTCTTTTTATTATTTAGTGTCTTTTAATTTGGAGTCATATATAGCTAGTCAGTGTCTTGTAATGAAACTTTGGTTGTTGCTTCTTCAACA
  clone4-3 ACTAGTTTGATCCTGTTTTGTATTTTGTATCGATCTGTGTTTCTTTTTATTATTTAGTGTCTTTTAATTTGGAGTCATATATAGCTAGTCAGTGTCTTGTAATGAAACTTTGGTTGTTGCTTCTTCAACA
 clone4-2b ACTAGTTTGATCCTGTTTTGTATTTTGTATCGATCTGTGTTTCTTTTTATTATTTAGTGTCTTTTAATTTGGAGTCATATATAGCTAGTCAGTGTCTTGTAATGAAACTTTGGTTGTTGCTTCTTCAACA
 clone4-1b ACTAGTTTGATCCTGTTTTGTATTTTGTATCGATCTGTGTTTCTTTTTATTATTTAGTGTCTTTTAATTTGGAGTCATATATAGCTAGTCAGTGTCTTGTAATGAAACTTTGGTTGTTGCTTCTTCAACA
  clone4-5 ACTAGTTTGATCCTGTTTTGTATTTTGTATCGATCTGTGTTTCTTTTTATTATTTAGTGTCTTTTAATTTGGAGTCATATATAGCTAGTCAGTGTCTTGTAATGAAACTTTGGTTGTTGCTTCTTCAACA
 clone4-3b ACTAGTTTGATCCTGTTTTGTATTTTGTATCGATCTGTGTTTCTTTTTATTATTTAGTGTCTTTTAATTTGGAGTCATATATAGCTAGTCAGTGTCTTGTAATGAAACTTTGGTTGTTGCTTCTTCAACA
 Consensus ACTAGTTTGATCCTGTTTTGTATTTTGTATCGATCTGTGTTTCTTTTTATTATTTAGTGTCTTTTAATTTGGAGTCATATATAGCTAGTCAGTGTCTTGTAATGAAACTTTGGTTGTTGCTTCTTCAACA

          651    66062
          |--------+-|
  clone4-1 ATCCAGTCTTCA
  clone4-2 ATCCAGTCTTCA
 clone4-16 ATCCAGTCTTCA
 clone4-15 ATCCAGTCTTCA
 clone4-14 ATCCAGTCTTCA
 clone4-13 ATCCAGTCTTCA
 clone4-12 ATCCAGTCTTCA
 clone4-11 ATCCAGTCTTCA
 clone4-10 ATCCAGTCTTCA
  clone4-9 ATCCAGTCTTCA
  clone4-8 ATCCAGTCTTCA
  clone4-6 ATCCAGTCTTCA
 clone4-4b ATCCAGTCTTCA
  clone4-3 ATCCAGTCTTCA
 clone4-2b ATCCAGTCTTCA
 clone4-1b ATCCAGTCTTCA
  clone4-5 ATCCAGTCTTCA
 clone4-3b ATCCAGTCTTCA
 Consensus ATCCAGTCTTCA
```

Figure S3: Nucleotide sequence alignment of 18 cloned PGSC0003DMG400031236 cDNAs. The PGSC0003DMG400031236-encoded cDNA was amplified by PCR, cloned in pCR™4Blunt TOPO® vector (ThermoFisher Scientific), and the resulting construct was introduced into One Shot TOP10 *E. coli* cells (ThermoFisher Scientific). Eighteen kanamycin-resistant isolated colonies were then cultured in LB medium supplemented with 50 mg/l kanamycin. Plasmid DNA was extracted from each culture and sent for Sanger sequencing. Sequences alignment showed that PGSC0003DMG400031236 has four identical alleles in Premier Russet (one clone, 3b, had a 60-bp insertion that likely corresponds to an unspliced variant).
